# Type B Idiopathic Bone Defect of Mandible: An Etiopathogenic Dilemma

**DOI:** 10.1155/2012/482758

**Published:** 2012-05-30

**Authors:** Aakarsh V. Jhamb, Parul A. Jhamb, Aparna Dave, Vishwa Prakash Shetty

**Affiliations:** ^1^Department of Oral & Maxillofacial Surgery, ESIC Dental College, Rohini, New Delhi 110 089, India; ^2^Department of Oral & Maxillofacial Pathology, Santosh Dental College & Hospital, Ghaziabad 201 009, India; ^3^Department of Oral & Maxillofacial Pathology, SGT Dental College & Hospital, Gurgaon 122 505, India

## Abstract

Etiopathogenesis of the pathologic lesions forms the basis for formulation of appropriate intervention and further prevention. There is still a vast unknown field that has to be explored to know the causative reason behind certain benign & malignant lesions. Idiopathic bone defects are nonodontogenic pseudocystic cavities that are seen in the long bones & jaw bones. Radiographic interpretation is at times inadequate in diagnosis of odontogenic & nonodontogenic radiolucent lesions involving jaw bones. Histopathology has different criteria to segregate this lesion. In this paper, we discuss a case of type B histopathological variant of idiopathic bone defect that may suggest an alternative pathogenesis from type A variant.

## 1. Introduction

Radiolucent lesions in the jaw can occur in varied forms and may represent innocent anatomic variations, benign or malignant processes. Appropriate diagnosis relies on a detailed history, astute clinical sense, and cognition of the fact that pathology can present in unexpected forms. Cystic lesions are the most frequently encountered pathologic radiolucencies in the jaw [1]. This spectrum consists of pathological entities such as ameloblastoma, odontogenic keratocyst, and odontogenic myxoma. Most of these lesions cause symptomatic, expansile swelling that is quite obvious clinically; yet certain asymptomatic radiolucent pathologic lesions occur in the jaws that pose a significant problem in the diagnosis due to lack of definite objective or subjective clinical symptoms [2]. Idiopathic bone defect is one such intriguing lesion that occurs in the jaws relatively rarely. We elucidate a case of idiopathic bone defect of mandible with its postoperative course and discuss the pertinent review of literature. 

## 2. Report of a Case

**Figure 1:**
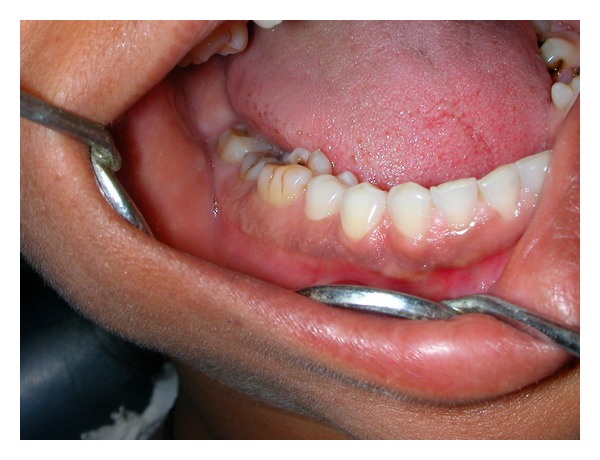
Intraoral view shows slight expansion of the buccal cortex in the region of mandibular first molar on right side.

A 35-years-old female visited our clinic in August 2009 with a complaint of swelling on right side of face, which had slowly increased in size over last one year before the patient approached for treatment. There was no history of trauma or any previous infection in the affected area. The past medical and surgical history was noncontributory. On physical examination the facial symmetry was maintained. Intraorally, slight expansion of the buccal cortex was seen in mandibular first molar region on right side (Figure 1). On palpation, the swelling was nontender, bony hard and localized over the alveolus of the mandibular first molar region on the buccal side. The teeth in the involved area were normal. There was no paresthesia or cervical lymphadenopathy.

**Figure 2:**
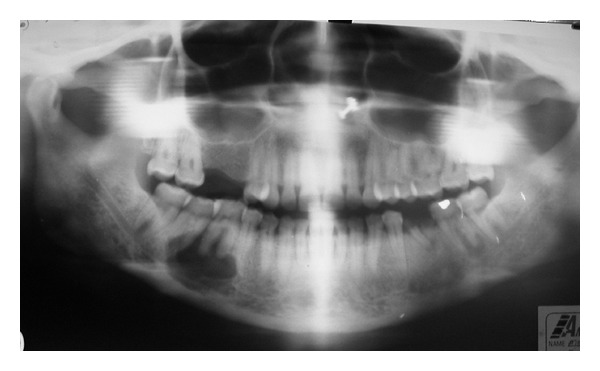
Orthopantomograph reveals a well-localized unilocular radiolucency in the mandibular first molar area on the right side involving the second premolar and second molar roots. Resorption of alveolar bone and loss of lamina dura around the first molar roots is also present.

Radiographic examination revealed a well-localized unilocular radiolucency in the mandibular first molar area on the right side involving the second premolar and second molar roots. The alveolar bone around the first molar roots was resorbed more than two-thirds of the root length, and there was loss of lamina dura around the associated vital teeth (Figure 2). Chest radiographs and blood chemistry were normal. Aspiration with a wide bore needle was attempted but yielded no results as it could not penetrate the radiolucent lesion.

**Figure 3:**
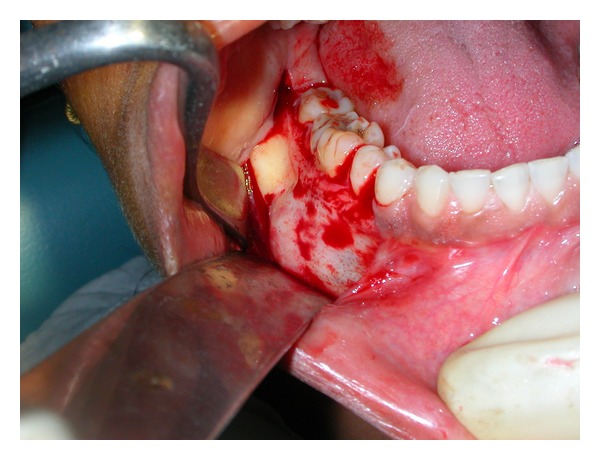
Intraoperative view shows expansion of the underlying buccal cortex with a bluish tinged surface is seen.

**Figure 4:**
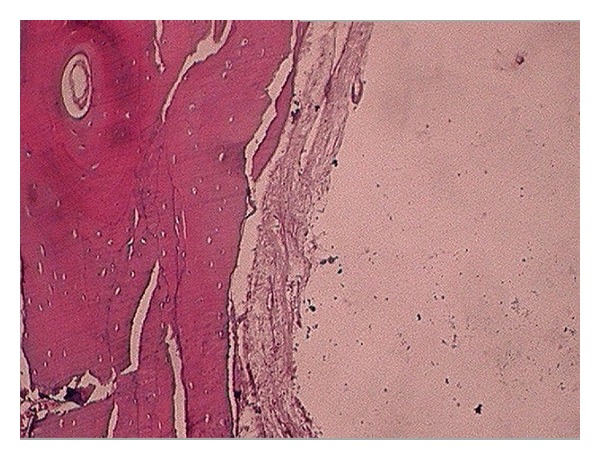
H&E stained section of the curetted tissue exhibits osseous cystic wall and overlying fibrovascular tissue with no epithelial lining. (10x).

**Figure 5:**
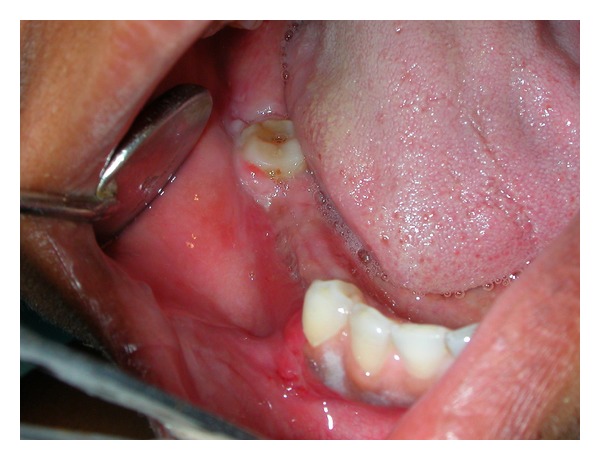
Clinical photograph of postoperative healed lesion.

**Figure 6:**
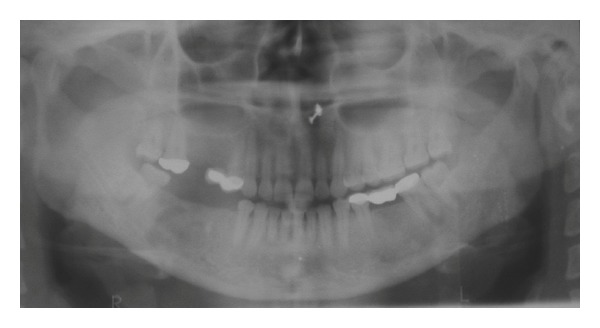
Orthopantamograph taken after 1 year shows complete bony healing.

A differential diagnosis of ameloblastoma, central giant cell granuloma, odontogenic keratocyst, and traumatic bone cyst was made. It was decided to take an incisional biopsy after extraction of the involved first molar under local anesthesia. Upon raising the mucoperiosteal flap, the underlying buccal cortex showed expansion and a bluish tinge (Figure 3). A small drill hole was made in the bone to allow insertion of a needle for aspiration. The bony window was then enlarged to take incisional biopsy. The bony cavity was found to be almost empty with scanty serosanguinous fluid. A provisional diagnosis of solitary bone cyst was made at the time of surgical exploration. The involved teeth were extracted and sufficient bleeding was induced in the cavity after curettage of the cavity walls. The wound was closed primarily with 3-0 silk sutures. Histopathological examination of the curetted tissue exhibited osseous cystic wall and overlying fibrovascular tissue with no epithelial lining (Figure 4). A thick fibrovascular wall with underlying dysplastic bone could be appreciated which was suggestive of type B simple bone cyst [3]. Postoperatively, the wound healed uneventfully (Figure 5). The patient was followed up for next one year, as type B lesion has chances of recurrence when compared with type A. After 1 year the radiographs showed complete bony healing (Figure 6).

## 3. Discussion

Idiopathic bone defect has been referred by various names: hemorrhagic cyst, traumatic bone cyst, simple bone cyst, solitary bone cyst, unicameral bone cyst, progressive bone cavity, extravasation cyst [4, 5]. However, WHO has recommended the use of term solitary bone cyst since 1992 and in its more recent meeting in 2005 this entity has been classified under bone-related lesions [6]. It was also classified as a pseudocyst because it shows no epithelial lining. According to a recently proposed concept SBC is considered to be a synovial cyst arising from a juxtaepiphyseal error with intraosseous incorporation of synovial tissue [7]. As per the previous reports, development of this lesion has been attributed to the intrusion of salivary gland tissue, mechanical pressure of the surrounding tissue, and the pressure of the facial artery as the most probable factors [8]. It has been classified by different authors as either (i) stafne type (ii) cystic type, or (i) type A, (ii) type B on radiographic and histopathological assessment [3]. Matsumura et al. stated that type B lesion results from cystic degeneration of certain type of fibroosseous lesions and type A might be caused by intramedullary hemorrhage and its sequelae. Most of the type B cysts are known to displace mandibular canal and cause buccolingual expansion in comparison with the contralateral arch as seen in this case [3]. 

This lesion presents mostly as an osteolytic lesion and forms a cavity with either a geodic or polymorphous shape [9]. It may be empty or filled with blood, serum, or serosanguinous fluid. No cholesterol crystals have been reported and with time the amount of fluid diminishes [2]. This lesion is mainly diagnosed in young patients, mostly during the second decade of life, though in this case the lesion was diagnosed in late third decade. The sex distribution is reported to be quite even, although there is a male predominance for the extra facial variants [9, 10]. SBC evolution is asymptomatic in the beginning and is often discovered serendipitously during examination of a panoramic radiograph. Pain is the presenting symptom in 10 to 30% of patients and swelling has been reported in up to a fourth of the patients. Other symptoms include tooth sensitivity, paresthesia, fistula, and even pathologic fracture of the jaw bone [11]. As solitary bone cyst usually does not show any particular pathognomonic feature and the presentation is mostly a vague asymptomatic radiolucent shadow, so the definitive diagnosis can be made only upon surgical exploration and histopathological evaluation.

Radiographically, solitary bone cyst can have varied presentations as interradicular, periapical, pseudomultilocular, solitary cyst-like contacting teeth and solitary cyst-like not necessarily contacting teeth. Usually it appears as a well-defined, round-to-ovoid-cyst-like radiolucency above the mandibular canal [1]. The spectrum of differential diagnosis for such cases includes ameloblastoma, ameloblastic fibroma, central giant cell granuloma, odontogenic keratocyst, lateral radicular cyst, lateral periodontal cyst, vascular lesions, and ossifying fibroma. The segregation of this peculiar entity is through radiographic and histopathologic interpretation.

Ameloblastoma may present as a unilocular or multilocular expansile radiolucency in the jaws. The unilocular lesion can be confused primarily with a dentigerous cyst. It often causes smooth, regular root resorption, displacement of teeth as well as inferior alveolar canal that can differentiate it from solitary bone cyst.

 Although keratinizing cystic odontogenic tumor (KCOT) radiographically has a better defined outline, again it causes displacement and resorption of tooth apices unlike solitary bone cyst. Other odontogenic cysts such as lateral radicular cyst or lateral periodontal cysts are also potential considerations, during the diagnosis of SBC that occurs in the interradicular area of tooth-bearing regions of jaws. These lesions are associated with nonvital tooth; the radiolucencies are ovoid as against slit-like picture with smooth contours as seen in SBC [1]. The confusion related to cysts and tumors of odontogenic origin is ruled out by histopathological picture that is characteristic for these lesions.

The prognosis is usually better when the lesion is treated by fenestration or packing the cavity and the average time for complete healing after surgery is frequently between 1 to 1.5 years. The healing or recurrence can be confirmed within 3 years of treatment. The recurrence rate of solitary bone cyst is between 20 and 30% but it has been suggested that SBC cases associated with cemento osseous dysplasia or other lesions have a high probability of recurrence [12]. The rarity of this lesion in adults supports the hypothesis of spontaneous resolution.

 Cystic and cystic-appearing lesions that occur in the mandible are often difficult to distinguish on the basis of radiography alone. Therefore, these lesions must be aspirated, enucleated, and examined microscopically to establish accurate diagnosis. Solitary bone cyst is one such condition or, precisely, a misnomer that requires reevaluation as it may spontaneously resolve, may not have a history associated with trauma, and a definitive etiopathogenesis is still uncertain although histopathological typing may be suggestive of a particular causative factor for this defect.
